# Identification of inversion domains in KTiOPO_4_ *via* resonant X-ray diffraction

**DOI:** 10.1107/S2053273315007238

**Published:** 2015-05-14

**Authors:** Federica Fabrizi, Pamela A. Thomas, Gareth Nisbet, Stephen P. Collins

**Affiliations:** a Diamond Light Source, Harwell Science and Innovation Campus, Didcot, OX11 0DE, England; b Department of Physics, University of Warwick, Coventry, CV4 7AL, England

**Keywords:** resonant X-ray diffraction, synchrotron radiation, imaging, absolute structure, inversion symmetry, inversion domains, ferroelectrics

## Abstract

A novel method is presented for the identification of the absolute crystallographic structure in multi-domain polar materials such as ferroelectric KTiOPO_4_. Resonant (or ‘anomalous’) X-ray diffraction spectra collected across the absorption *K* edge of Ti (4.966 keV) on a single Bragg reflection demonstrate a huge intensity ratio above and below the edge, providing a polar domain contrast of ∼270. This allows one to map the spatial domain distribution in a periodically inverted sample, with a resolution of ∼1 µm achieved with a microfocused beam. This non-contact, non-destructive technique is well suited for samples of large dimensions (in contrast with traditional resonant X-ray methods based on diffraction from Friedel pairs), and its potential is particularly relevant in the context of physical phenomena connected with an absence of inversion symmetry, which require characterization of the underlying absolute atomic structure (such as in the case of magnetoelectric coupling and multiferroics).

## Abstract 2

The identification and high-resolution mapping of the absolute crystallographic structure in multi-domain ferroelectric KTiOPO_4_ is achieved through a novel synchrotron X-ray diffraction method. On a single Bragg reflection, the intensity ratio in resonant diffraction below and above the Ti absorption *K* edge demonstrates a domain contrast up to a factor of ∼270, thus implementing a non-contact, non-destructive imaging technique with micrometre spatial resolution, applicable to samples of arbitrarily large dimensions.

## 1. Introduction   

Modern condensed matter physics is increasingly concerned with phenomena that require an absence of inversion symmetry, either locally (local atomic environment) or globally (crystal point group). For example, multiferroic materials often exhibit magnetic structures that are either driven by the underlying low symmetry of the crystal structure, or lead to a spontaneous symmetry breaking, often giving rise to strong magnetoelectric coupling. However, in relating the spin structures to the underlying atomic ordering, the latter must be characterized with equal confidence. One type of structure of interest is represented by polar systems and ferroelectrics.

Among the crystal classes lacking inversion symmetry, those possessing at least a direction whose two senses are geometrically or physically different (polar direction) are called piezoelectric; among those, pyroelectric crystal classes possess a polar direction which has no symmetrically equivalent directions, and are therefore capable of sustaining a permanent electric dipole moment. Such systems are energetically degenerate (in the absence of external fields) with their spatially inverted counterparts, leading to the possibility of multi-domain states. Ferroelectrics are a subset of pyroelectric crystals whose defining property is the ability to switch the sense of spontaneous polarization upon application of an electric field of magnitude greater than the coercive field. Piezoelectric crystals also exhibit nonlinear optical susceptibility leading to second-harmonic generation: monochromatic light waves passing through the crystal induce new waves of twice the incident frequency. A way to exploit this feature for a variety of applications is the quasi-phase-matching technique, implemented through the modulation of the second-harmonic coefficient along the direction of propagation. This can be obtained by preparing a ferroelectric crystal in a state of periodically spaced domains of alternating polarity, *via* the application of an external electric field using patterned electrodes. Materials such as KTiOPO_4_ (KTP), LiNbO_3_ and LiTaO_3_ can offer very good conversion efficiencies for any desired frequency within their transparency range.

A variety of experimental techniques to reveal the integrity of the domain periodicity and the quality of the resulting crystal have been traditionally employed (Soergel, 2005 ▸; Potnis *et al.*, 2011 ▸). Selective etching combined with optical microscopy is fast and simple and provides strong evidence of ferroelectricity, but is destructive to the sample; transmission electron microscopy (TEM) and scanning electron microscopy (SEM) offer sub-micron resolution, but at the expense of potentially altering the domain configuration *via* the electron beam, besides the constraints placed on sample thickness in the case of TEM. Surface-sensitive techniques such as atomic force microscopy and electrostatic force microscopy are also employed, although the results may be difficult to interpret.

All-optical imaging and wide-field microscopy techniques such as optical polarizing microscopy, light-deflection near-field, interference and birefringence techniques and photorefractive techniques have the advantage of being non-contact, non-invasive methods allowing lateral resolution of the order of 1 µm, but they require the application of an external voltage or sophisticated pump-and-probe setups if one wishes to differentiate between antiparallel polar domains. Closely related to these are X-ray diffraction topography techniques, which also provide a one-shot mapping of the domain spatial distribution and offer resolution up to ∼1 µm depending on the detector. The contrast mechanisms are based on properties secondary to the polarization direction, such as the inhomogeneous strain between antiparallel domains introduced during the poling process (Hu *et al.*, 1995 ▸, 1996 ▸, 1999 ▸) or the strain at the domain walls (Kim *et al.*, 2000 ▸; Jach *et al.*, 2004 ▸). The method can be enhanced with an experimental setup that is sensitive to the phase shift between waves diffracted from different regions of the crystal, by combining Fresnel phase imaging with Bragg diffraction imaging (Pernot-Rejmánková *et al.*, 2000 ▸, 2003 ▸; Rejmánková-Pernot *et al.*, 1998 ▸). Even if it introduces a further level of complexity in the interpretation of the results, this technique provides access to the phase shifts between the structure factors of opposite domains in a multi-domain sample (Hu *et al.*, 1998 ▸; Soergel, 2005 ▸). Since the structure factors are directly dependent on the atomic positions, the contrast mechanism is anchored to the crystallographic configuration. The absolute orientation of each domain cannot be determined from the phase shifts, but these can be used to reveal information about the relative atomic positions between domains, for instance to identify the pivot atoms responsible for the domain matching at the walls.

In this work we introduce a technique based on resonant (or ‘anomalous’) X-ray diffraction (RXD) which, in contrast to those outlined above, combines the features of being non-destructive and non-invasive with the further advantage of directly probing the squared structure factor of the diffraction cross section, and therefore of being related to the atomic positions, without the need for a phase-sensitive measurement apparatus. This technique measures the domain-specific intensity of the resonant structure factor in the material under investigation, rather than the phase shifts and interference between waves diffracted from opposite domains. As such, it is able to determine the absolute orientation in a sample with any arbitrary pattern of either single or multiple domains. Moreover, our approach is different from traditional resonant X-ray techniques in that it only requires diffraction profiles from a single Bragg reflection and is applicable to crystals of arbitrarily large dimensions; therefore, we suggest that this method can be established as a valuable tool in the characterization of those materials whose absolute structure plays an important role in the setting of physical properties such as the magnetoelectric effect and multiferroicity. By mapping the inversion domains in large crystals to establish their monodomain nature or obtain a spatial image of the domains, the information thus acquired can be combined, for example, with chiral spin structure data to obtain insight about the exchange interactions responsible for the magnetic structure and magnetoelectric coupling.

In the traditional resonant X-ray diffraction technique for determining the absolute structure of a crystal, the contrast mechanism is provided by the resonant contribution to the atomic scattering factor of a specific element becoming enhanced in proximity to an X-ray absorption edge, adding a complex and energy-dependent coefficient to the real form factor 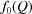: 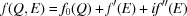. The structure factor of a non-centrosymmetric compound may then generate a domain-dependent difference in the intensities of two Friedel reflection pairs (related by inversion of the scattering vector 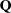) when the photon energy *E* is sufficiently close to an absorption edge of one of the chemical species of the compound (Bijvoet, 1954 ▸). By refining the intensities of a large set of reflections comprising numerous Friedel pairs, the spatial arrangement of the atoms of a non-centrosymmetric crystal can be determined without ambiguity between the two inversion-related images. To this end, the procedure introduced by Flack (1983 ▸) has improved on previously used methods (Hamilton, 1965 ▸; Rogers, 1981 ▸), and has since established itself as the main standard technique (Flack & Bernardinelli, 1999 ▸, 2000 ▸, 2008 ▸). In this method, any non-centrosymmetric crystal is treated as a twin by inversion, and the so-called Flack parameter is defined as the fractional contribution to the diffraction of one of the twins, thus ranging from 0 to 1. The parameter is then considered as variable during the least-squares refinement of the crystal structure, alongside the atomic coordinates. In those cases in which the basic crystal structure is known and only the inversion domain is to be determined, similar to the case that we present here, other approaches have sought to restrict the measurement to those reflection pairs for which the discrepancy from the Friedel law is more pronounced (Page *et al.*, 1990 ▸; Grochowski, 1997 ▸; Grochowski & Serda, 1997 ▸). Unfortunately, these methods presuppose collecting the intensities of reflections opposite to each other from the same scattering volume, which may not be feasible in crystals whose thickness is several mm, due to beam attenuation; even in the case of small crystals, the refinement required by Flack & Bernardinelli (1999 ▸) relies on an often subtle contrast between diffraction intensities in different geometries, that may be influenced by shape effects and be susceptible to systematic errors due to absorption or anisotropic extinction. The novelty of our approach resides in measuring diffraction intensity *versus* energy from a single reflection around a suitable absorption edge: the contrast in intensity arising from different domains is introduced in the structure factor through the characteristic energy dependence of the resonant contributions. The spectra are interpreted with the aid of simulations developed using the tabulated anomalous scattering coefficients for the resonant atom (Waasmaier & Kirfel, 1995 ▸; Sasaki, 1989 ▸), and assisted by the software tools provided by the crystallographic library *CCTBX* (Grosse-Kunstleve *et al.*, 2002 ▸; Gildea *et al.*, 2011 ▸). By extracting the intensity ratio at appropriate energies above and below the edge, we show that the fraction of ferroelectric domains on the crystal surface can be determined and mapped. While our approach relies on the same physical principle as the traditional technique outlined above, and is not concerned with solving the crystal structure but merely with discriminating between inversion images of an already known structure, it is capable of making full use of the energy tunability provided by the synchrotron source to maximize the contrast between domains in the ratio of intensities collected at two different energies. As a consequence, one single reflection is sufficient for the task, and with a much higher sensitivity than offered by the refinement of Flack & Bernardinelli (1999 ▸), provided that the Miller indexes are suitably chosen. By eliminating the need to collect opposite reflection pairs, crystals of arbitrary thickness can be characterized; this in turn enables us to avoid shape and size effects, and to take advantage of the simple scattering geometry of a large, well defined and fixed surface.

## 2. Experimental details   

The KTP sample is the same as used in Lyford *et al.* (2015 ▸); it is a plate whose dimensions are 8 × 4 mm and whose thickness is 0.4 mm. A small area has been low-temperature *a*-poled (domain walls parallel to the *bc* plane) with a grating period of Λ = 9.02 µm. In this standard bulk poling technique (Myers *et al.*, 1995 ▸; Soergel, 2005 ▸), the domain reversal is achieved through application of an external electric field: significant domain reversal occurs when the field, applied along the axis of spontaneous polarization, exceeds a certain value referred to as the coercive field. The desired domain configuration is precisely defined by the structure of the positive electrode, patterned by lithography on one surface of the sample. This yields uniform periodic polarization in a thick plate, with straight, vertical domain walls throughout the material volume. This fabrication method can achieve periodic structures on a scale of several cm with good reproducibility, while preserving the material’s transparency and optical non­linearity. After the poling, the sample was polished to a high optical quality to remove surface scratches.

The experiment has been carried out at the Materials and Magnetism beamline I16 at Diamond Light Source, making use of its microfocus capability. The energy of the incident radiation was tuned at the Ti *K* edge (4.966 keV) by means of a U27 undulator insertion device and a channel-cut Si(111) monochromator, while the sample was mounted on a Newport six-circle kappa diffractometer at ambient temperature, equipped with translation motors to perform raster scans over the sample surface. A Pilatus 100K photon-counting area detector was used to collect the diffracted radiation. In order to maximize lateral resolution in the high-resolution domain imaging measurement, a micron-sized focal spot is required: this was achieved by means of a Kirkpatrick–Baez (KB) mirror system mounted on the diffractometer. This assembly allowed us to reach a beam spot size of 1.2 µm (vertically) × 1.5 µm (horizontally).

## 3. Results and discussion   

**Figure 1:**
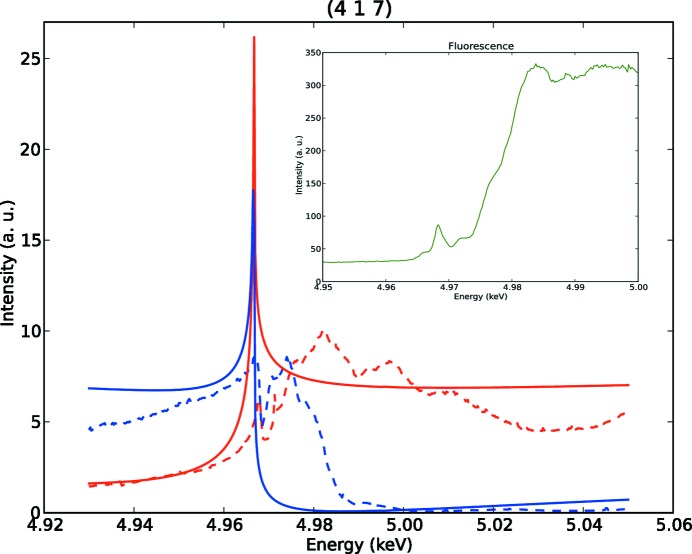
Dashed lines: energy dependence of the diffraction intensity on the reflection (417) across the Ti *K* edge collected on the two opposite surfaces of the sample, configuration ‘up’ (red) and ‘down’ (blue). Solid lines: energy dependence calculated from simulations of a monodomain crystal of domain ‘*A*’ (blue) and ‘*B*’ (red), in which domains *A* and *B* are related by inversion. Inset: the measured fluorescence spectrum. The statistical errors for all intensity measurements are smaller than the line symbols.

The incident-beam energy was tuned to the Ti *K* edge, as shown in the fluorescence scan across the absorption edge in the inset of Fig. 1 ▸.

From the simulations, it was apparent that the reflection (417) is particularly sensitive to the domain composition. The reason for this can be explained as follows. The ultimate aim of the measurement is to discriminate between domains from the intensities collected at two fixed energies (above and below the edge). The ideal situation is one in which a significant intensity contrast between domains exists at an energy below the edge, and this contrast remains significant but reverses its sign at an energy above the edge. In this situation, the contrast is strong at both fixed energies and changes dramatically with energy across the edge. The former makes the ratio of intensities below and above the edge highly sensitive to the domain composition, while the latter allows domain orientation to be determined even when the total signal is highly inhomogeneous. The structure factor contributing to the intensity of reflection 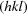 from a single domain can be expressed as 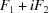, where 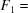 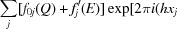 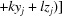 collects the contributions from the Thomson and real anomalous scattering factors of the *j* atoms at positions 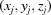 in the unit cell, and 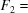 
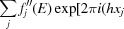
 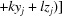 collects the anomalous imaginary scattering factors. The energy dependence of both 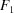 and 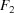 in the range of interest is essentially due to the resonant Ti atoms. At fixed energy, the intensity contrast between the two inversion domains ‘*A*’ and ‘*B*’ is 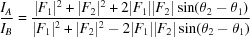
where 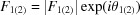 are defined as the factors for domain ‘*A*’. Therefore the requirement outlined above implies that 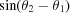, arising from the phase shift between 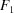 and 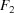, must be reasonably strong at both energies, and reverse its sign between them. In the case of reflection (417), 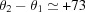
° at *E* = 5.007 keV above the edge, and 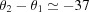
° at *E* = 4.96 keV below the edge, thus satisfying all conditions.

**Figure 2:**
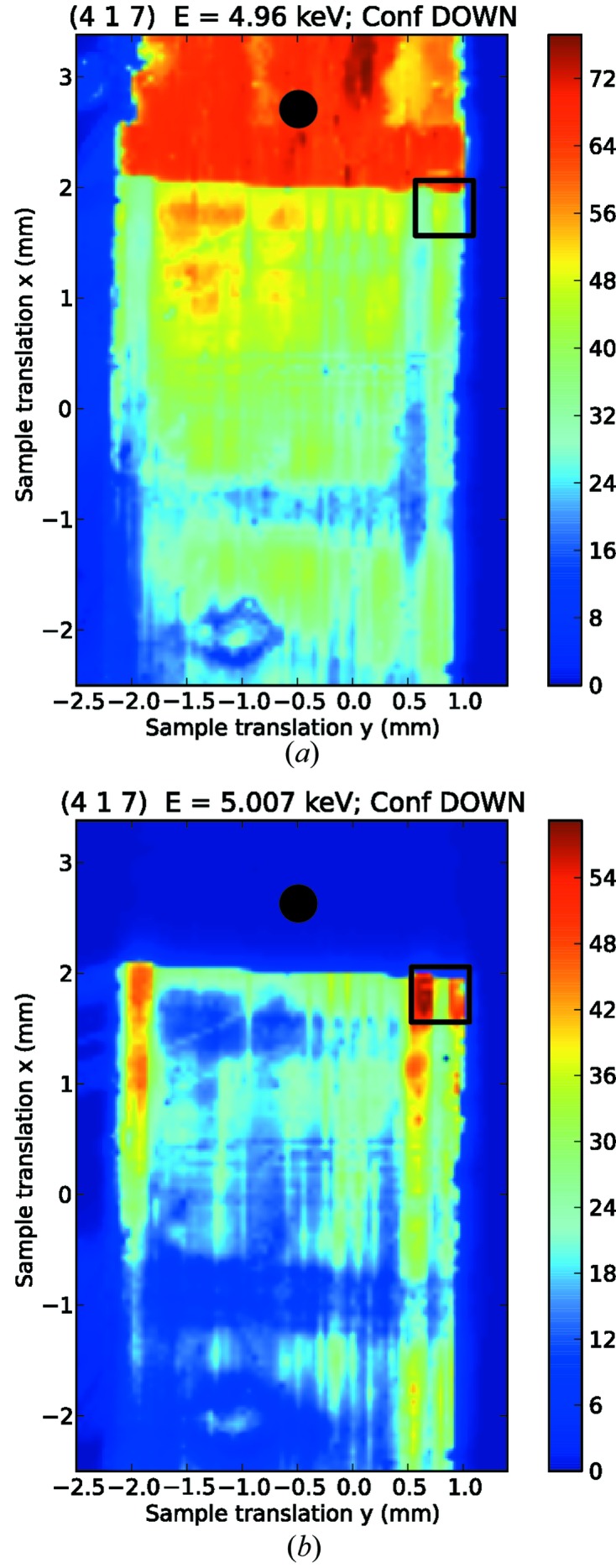
Low-resolution maps of the diffraction intensity on the reflection (417) at (*a*) *E* = 4.96 keV and (*b*) *E* = 5.007 keV (beam spot ∼ 0.015 × 0.05 mm), measured on the same surface of the sample (configuration ‘down’). The energy spectra in Fig. 1 ▸ have been collected in the monodomain region in the top part of the sample (black dots), while the high-resolution maps in Fig. 3 ▸ have been collected in the periodically domain-inverted region (enclosed in black squares).

A low-resolution map of the spatial distribution of domains was obtained by rastering the sample aligned on said reflection at the two different energies below and above the edge (*E* = 4.96 and 5.007 keV), where the energy above the edge has been specifically chosen to correspond to an intensity of almost zero, in order to enhance the contrast between domains. At this stage the beam was defocused (with both main mirrors and KB mirrors out) and the beam spot was defined by the sample slits to be 0.015 × 0.05 mm. The results are plotted in Fig. 2 ▸. The main feature to be observed is that there is a large monodomain region on the top part of the sample, which can be appreciated by its homogeneous intensity and the contrast above and below the edge.

Therefore, as a first step, the detailed energy dependencies have been collected in the monodomain region for the reflection (417). As a way of confirming the validity of the technique, this measurement has been repeated on the two opposite surfaces of the sample, an operation that, given the symmetry of the crystal, is equivalent to collecting data on the same surface from the two opposite polar domains. Given the prevalence of multiple scattering peaks, the energy spectra have been repeated at three different azimuth angles, spaced by 1°, and averaged together with the exclusion of the outlying data points, in order to give a reliable profile. The results are plotted in Fig. 1 ▸ (dashed lines): the two energy spectra show remarkable differences, that can be interpreted as resonant scattering contributions with the aid of the simulation tools outlined above. The two crystallographic domains are assumed to be related by spatial inversion, and the results corrected by absorption are plotted as solid lines against the data in Fig. 1 ▸. The calculations are not expected to capture the details across the edge that are specific to the material. We note that the diffraction anomalous fine structure (DAFS) oscillations apparent in the energy scan in Fig. 1 ▸ contain information about the electronic structure and the local atomic environment at the Ti sites. These are not reflected in our simple model, which has been designed to be adequate for the goal of domain discrimination, and is based on the anomalous scattering coefficients of the isolated ions. As expected, there is however a reasonable agreement at the energies immediately above and below the absorption edge. The pronounced contrast offered by the technique is apparent in the intensity ratio below/above the edge for one domain being ∼270 times the corresponding ratio for the inverted domain. This allowed us to restrict the measurement to only two points in energy, from which sufficient sensitivity to quantitatively assess the domain fraction is achieved.

**Figure 3:**
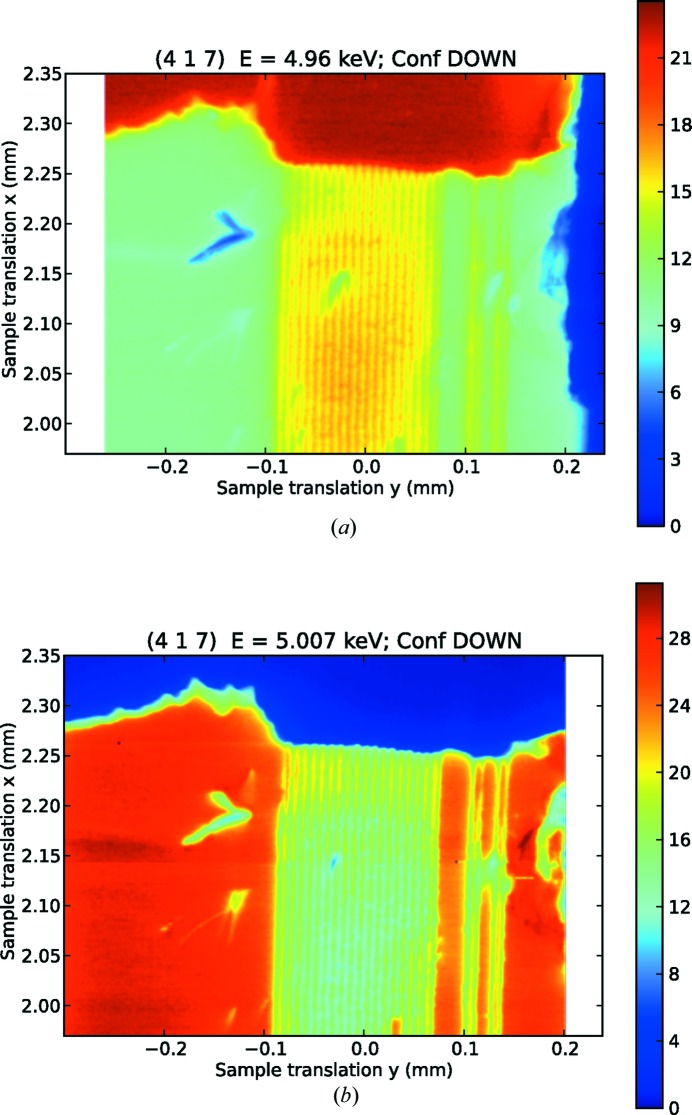
High-resolution maps on the periodically domain-inverted region at (*a*) *E* = 4.96 keV and (*b*) *E* = 5.007 keV (beam spot ∼ 1.2 × 1.5 µm achieved with microfocusing), collected on the same surface of the sample.

**Figure 4:**
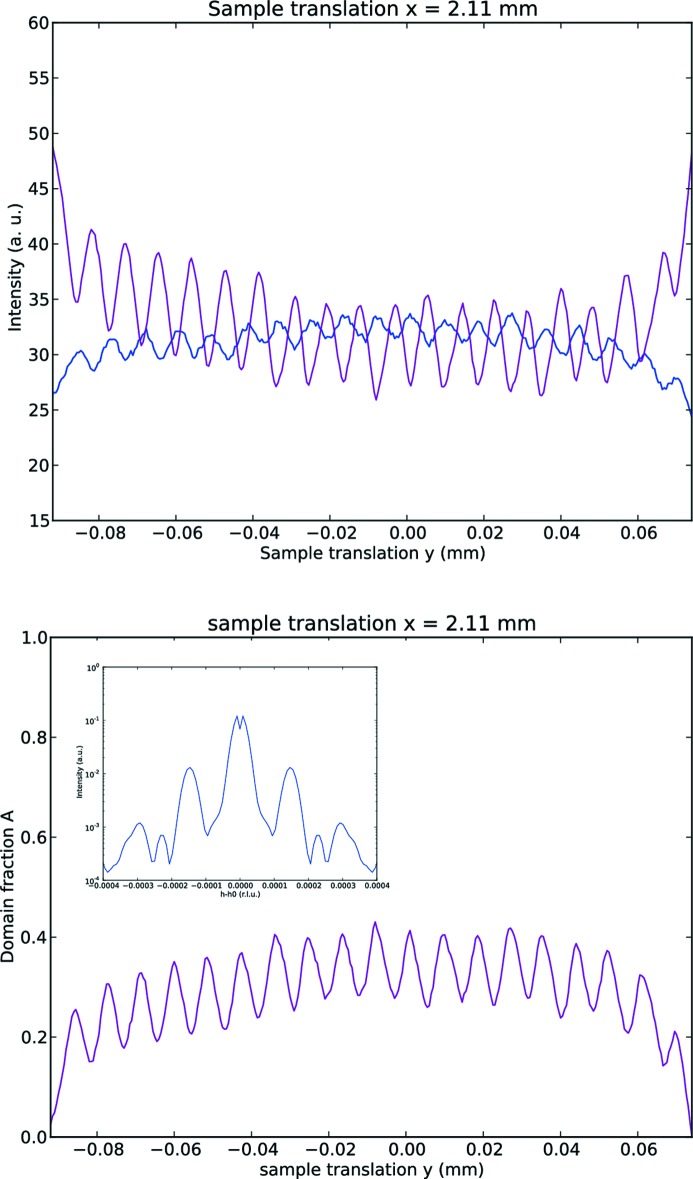
(*a*) One-dimensional translation scans of the diffraction intensity along the *a* axis across the periodically domain-inverted region, at energies *E* = 4.96 and 5.007 keV; note that the intensities below and above the edge are modulated in antiphase to each other. (*b*) Domain fraction (from 0 = domain ‘*B*’ to 1 = domain ‘*A*’) extracted from the ratio of intensities above. Inset: reconstruction of the (417) satellite reflections in reciprocal space induced by the periodic modulation of the domain fraction calculated above. The intensity (in log scale) is plotted against the shift in position along a* relative to the central value 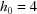.

**Figure 5:**
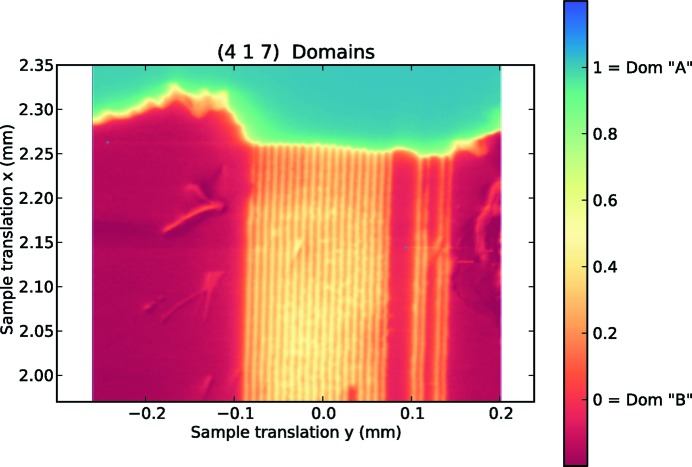
High-resolution map of the domain fraction (from 0 = domain ‘*B*’ to 1 = domain ‘*A*’) extracted from the ratio of intensities plotted in Fig. 3 ▸.

Since the experimental capability to distinguish between the crystallographic domains had been established, we proceeded to identify and map the surface area artificially poled by the electric field. To this end, we have made use of the microfocusing provided by the KB mirrors. The same procedure used in the case of the low-resolution map has been applied, rastering the sample aligned on the reflection (417) at the same energies below and above the edge (*E* = 4.96 and 5.007 keV). The results are in Fig. 3 ▸, and the alternating pattern of domains is clearly visible (we note that the patterns measured at the two photon energies are shifted slightly due to the microfocused beam not being aligned to the centre of rotation). The orientation of the sample that brings the crystal into diffraction condition determines a beam footprint on the surface of 1.5 µm (along the direction indicated in the figure as translation *x*) × 1.75 µm (along the direction *y*). The stability of the microfocused beam has been characterized, resulting in a drift in position which is largely linear and of the order of a few microns per 12 h, roughly corresponding to the time necessary to acquire one map. The drift in position is corrected when extracting the point-by-point ratio between intensities from the two different maps, as part of the overall shift that had to be applied to correct for the centre of rotation. To obtain a quantitative estimate of the purity of the domains induced by the electric poling, we have collected a series of one-dimensional translation scans that cut through the *a*-poled domains along the direction 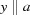, for the two energies and for three different positions in the orthogonal direction *x*. One of these scans is plotted in Fig. 4 ▸(*a*). The corresponding domain fraction profile has been extracted by comparison with the simulation of the sum of two incoherent diffraction domains ‘*A*’ and ‘*B*’ related by inversion, to which end the ratio of intensities between energies below and above the edge has been employed: 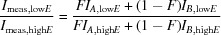
where *F* is the domain fraction (from 0 = domain ‘*B*’ to 1 = domain ‘*A*’), 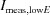 and 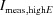 are the intensities from the one-dimensional cuts at the two energies, and 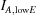 (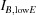) and 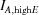 (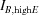) are the intensities from the simulations for domain ‘*A*’ (‘*B*’) at the two energies. The results for this specific scan cutting across the domains (Fig. 4 ▸ *b*) indicate that the fraction of crystallographic domain ‘*A*’ ranges from ∼0.18 to ∼0.4. This same procedure has been extended to the whole high-resolution map, thus obtaining the complete two-dimensional pattern of domains in Fig. 5 ▸.

This information can be compared with the results obtained on the same sample in Lyford *et al.* (2015 ▸) by means of reciprocal-space mappings, a well established technique for the study of periodic arrangements of polarization domains (Zubko *et al.*, 2010 ▸; Catalan *et al.*, 2006 ▸). In our experiment, we have operated with a microfocused beam and positioned ourselves in reciprocal space on top of an integer-indexed Bragg reflection. We have then reconstructed the domain fraction 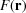 point by point in real space: specifically, we have extracted a profile 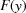 along the direction of the periodic modulation of domains (Fig. 4 ▸ *b*). In the experiment by Lyford *et al.* (2015 ▸), the beam has a larger footprint and the beam coherence significantly exceeds the period of the domain-inversion grating: the coherence length in Bragg geometry, calculated as a combination of the spatial (lateral) and temporal (longitudinal) coherence functions, is close to the value of 21.8 µm/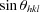, thus enabling the diffraction to capture the whole periodic modulation at once. At non-resonant photon wavelength, the periodic phase contrast between domains gives rise to satellite reflections around the central Bragg reflection, separated by 1/Λ (Λ being the period of the modulation), that have been mapped with high resolution in reciprocal space. Relating the two experiments, we calculate a Fourier transform of our measured 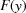 profile, multiplied by a Gaussian function in *y* simulating the illumination from a partially coherent beam (inset in Fig. 4 ▸ *b*). Since we obtain a semi-sinusoidal function for the domain modulation, the main features of the satellite distribution appear as one constant term (the central Bragg reflection), one main harmonic separated from the main reflection by 1/*N* reciprocal-space units (*N* being the number of crystallographic cells along the *a* direction enclosed in one period Λ), plus much weaker contributions from the finer details of the modulation. Comparing this with the analysis in Lyford *et al.* (2015 ▸), it can be seen that the two methods provide consistent and complementary results: the high-resolution reciprocal-space map offers a more detailed picture of the subtle deviations from a perfectly sinusoidal pattern, and is therefore able to provide additional information to the real-space mapping, while being a technique more limited in scope, as it requires the presence of both opposite domains in a periodical arrangement.

## 4. Conclusions   

In the case of periodically domain-inverted KTP, the energy dependence of the resonant X-ray diffraction from a single Bragg reflection has been exploited to determine the absolute crystallographic structure. The intensity ratio between two energy points below and above the Ti *K* edge, when measured on opposite surfaces of the sample to emulate diffraction from opposite monodomains, displayed domain contrast up to a factor of ∼270. The finely tuned sensitivity provided by this contrast has been exploited to obtain a µm-resolution image of the spatial distribution of domains, and to extract the domain fraction by comparison with a simple diffraction model based on the tabulated scattering coefficients of the resonant atom.

The quality of the sample under investigation has been thoroughly characterized in its periodically domain-inverted region, resulting in a domain fraction modulated as a sinusoidal curve varying within a range of about 20%, within our spatial resolution; this allows us to assess the efficacy of the electric poling as quite inferior to the ideal complete domain switching.

The method introduced in this paper is of considerable potential, being applicable to samples in a state of either single or multiple domains in an arbitrary pattern and, equally importantly, of arbitrary dimensions, which confers it a significant advantage over other long-established techniques dependent on resonant X-ray scattering. A promising line of work in this respect is the crystallographic characterization necessary to the comprehension of those magnetic phenomena that are closely connected to the non-centrosymmetric nature of the underlying atomic structure, such as displayed in multiferroic materials. The contrast mechanism, exploited here in a scanning microscopy setup, is also well suited for development in full-field imaging techniques such as topography, thus enabling one to perform real-time characterization of domain dynamics, for instance to follow domain evolution under applied external fields.
